# Findings in percutaneous trans-hepatic cholecysto-cholangiography in neonates and infants presenting with conjugated hyperbilirubinemia: emphasis on differential diagnosis and cholangiographic patterns

**DOI:** 10.1186/s12887-022-03816-y

**Published:** 2023-01-14

**Authors:** Dimitri A. Parra, Shannon E. Peters, Ruhail Kohli, Racha Chamlati, Bairbre L. Connolly, Justyna M. Wolinska, Vicky L. Ng, Michael J. Temple, Philip R. John, Binita M. Kamath, Simon C. Ling, Annie Fecteau, Afsaneh Amirabadi, Joao G. Amaral

**Affiliations:** 1 Division of Image Guided therapy, Department of Diagnostic Imaging, The Hospital for Sick Children, University of Toronto, Toronto, ON Canada; 2 Department of Family & Community Medicine, University of Toronto, Toronto, ON Canada; 3 Division of Gastroenterology and Hepatology, Johns Hopkins University School of Medicine, Baltimore, MD USA; 4 Department of Psychiatry, St. Elizabeth’s Medical Center, Brighton, MA USA; 5 Division of Pediatric Surgery, Children’s Hospital of Eastern Ontario (CHEO), Ottawa, ON Canada; 6 Division of Gastroenterology, Hepatology, and Nutrition, The Hospital for Sick Children, University of Toronto, Toronto, ON Canada; 7 Division of General and Thoracic Surgery, The Hospital for Sick Children, University of Toronto, Toronto, ON Canada

**Keywords:** Biliary atresia, PTCC, Pediatric cholangiography, Conjugated hyperbilirubinemia, Alagille’s syndrome

## Abstract

**Background:**

Biliary atresia (BA) is one of the causes of conjugated hyperbilirubinemia in infants which if untreated leads to end-stage liver disease and death. Percutaneous Trans-hepatic Cholecysto-Cholangiography (PTCC) is a minimally invasive study which can be utilized in the diagnostic work-up of these patients. This study’s purpose is to describe the experience with PTCC in neonates, the imaging findings encountered, and the abnormal patterns which warrant further investigation.

**Methods:**

A 16-year single-center retrospective study of patients with persistent neonatal cholestasis (suspected BA) undergoing PTCC. Patient demographics, laboratory values, PTCC images, pathology and surgical reports were reviewed.

**Results:**

73 patients underwent PTCC (68% male, mean age 8.7 weeks, mean weight 4.0 Kg). The majority of studies were normal (55%). Abnormal patterns were identified in 33 cases, 79% were diagnosed with BA and 12% with Alagille syndrome. Non-opacification of the common hepatic duct with a narrowed common bile duct (42%) and isolated small gallbladder (38%) were the most common patterns in BA.

**Conclusion:**

PTCC is a minimally invasive study in the diagnostic work-up of infants presenting with conjugated hyperbilirubinemia (suspected BA). Further invasive investigations or surgery can be avoided when results are normal.

## Introduction

Biliary Atresia (BA) is a rare disease of unknown etiology affecting neonates and infants. It is characterized by inflammation, fibrosis, and obliteration of the intra and extra-hepatic portions of the biliary tree [1, 2]. In some cases, it may be due to defective morphogenesis of the bile ducts [1, 2]. The incidence of BA ranges from 1 in 5000 to 1 in 18,000 live births globally [2]. It is the most common cause of neonatal cholestasis and the most frequent indication for pediatric liver transplantation [1]. If untreated, BA leads to end stage liver disease, liver cirrhosis and death.

The definitive clinical diagnosis of BA may be difficult due to its non-specific clinical presentation of jaundice, acholic stools and conjugated hyperbilirubinemia [3]. Persistent jaundice after 2 weeks of age necessitates fractionated bilirubin levels analysis, and if conjugated hyperbilirubinemia is found, an abdominal ultrasound (US) scan, tests easily obtainable by a primary care provider [3].

Other conditions such as neonatal hepatitis, parenteral nutrition (PN)-associated liver disease (PNALD), and Alagille syndrome have similar presentations [3]. To help with early identification of patients with suspected BA, Chen et al have reported success using colored stool cards [4]. Based on laboratory tests, imaging findings, clinical history and physical examination, a liver biopsy with a percutaneous trans-hepatic cholecysto-cholangiography (PTCC) may be considered in the diagnostic work-up [5].

Liver biopsy can be diagnostic in cases of intrahepatic disease, demonstrating histologic signs of obstruction and prognostic factors (such as degree of fibrosis). However as early histologic findings of BA may be non-specific, false negative diagnoses may occur [3]. Intra-operative cholangiography (either open or laparoscopic) and histologic examination of the duct remnant remains the standard criterion for diagnosis or exclusion of BA [3, 6–9]. A Hepatobiliary Imino-Diacetic Acid (HIDA) scan is not routinely used in this center in the diagnostic work-up of this patient population as it has a high sensitivity but low specificity for the diagnosis of BA and current guidelines suggest a limited diagnostic role [3].

PTCC is a percutaneous procedure in which radio-opaque contrast is injected into the gallbladder (GB) under fluoroscopy to delineate the biliary tree [10–13]. It is a minimally invasive diagnostic alternative to intra-operative cholangiography. Timely and expedited BA diagnosis is essential to improve outcomes, as the earliest a Kasai procedure is performed, the better the prognosis for native liver survival [14, 15].

There are a limited number of studies focused on PTCC in this clinical context [10–13]. Due to the disease rarity, there is a paucity of published images, and patient cohorts are small.

The objectives of this study are to retrospectively analyze PTCC findings in a cohort of neonates and infants with conjugated hyperbilirubinemia and suspected BA, correlating the imaging with their final diagnosis; and to identify abnormal patterns which warrant further investigations, such as intra-operative cholangiography.

## Methods

### Study design

This was a Research Ethics Board approved retrospective observational study of patients with persistent neonatal cholestasis who underwent a successful PTCC in interventional radiology (IR) at a tertiary pediatric center (300–320 beds) from January 1, 2000, to February 29, 2016. Individual consent was waived for this review.

Data sources included ISYS (an in-house radiological database), the Electronic Patient Chart (EPC) system, and Picture and Archiving Communication System (PACS, GE Centricity). All PTCCs performed in the study period were included. Patients over 12 months of age, patients post- Kasai procedure or liver transplantation, or other cholangiography (not via GB, for other indications) were excluded.

All patients referred for PTCC had undergone clinical exam, laboratory testing and imaging investigations as guided by a pediatric hepatologist at our center. The indication for PTCC was to assess the anatomy and patency of the bile ducts, as part of the diagnostic work-up of cholestasis. Data collected included patient demographics (age, weight, gender), pre- and post-procedural biochemistry values, procedural details from PTCC with/without percutaneous liver biopsy; and surgical information (Kasai procedure, open liver biopsy, liver transplantation). Percutaneous trans-hepatic cholecysto-cholangiography images were correlated with pathology reports (percutaneous or open liver biopsies, excised GBs, or explanted livers). Clinical, radiological, pathological, and surgical findings were correlated with PTCC imaging patterns, to distinguish BA from other neonatal cholestatic causes, (e.g., PNALD, Alagille syndrome). Intra-procedure and post-procedure complications were classified as minor or major according to the Society for Interventional Radiology (SIR) guidelines [16].

### Statistical analysis

Descriptive analysis was performed. Laboratory values were statistically analyzed with SPSS software ((IBM SPSS Statistics for Windows, Version 26.0. Armonk, NY: IBM Corp.) using a t-test in cases of data normally distributed, and Mann-Whitney U tests in data not normally distributed. Levels of total bilirubin (normal 1–12 μmol/L), Aspartate Aminotransferase (AST: normal 20–67 U/L), Alanine Aminotransferase (ALT: normal 5–33 U/L) and Gamma-Glutamyl Transferase (GGT: normal 8–127 U/L) were compared between different patient groups, e.g. normal vs abnormal PTCC; normal vs BA, Alagille syndrome, other diagnoses. A *p*-value < 0.05 (two-sided) was considered statistically significant.

### Procedure technique

**Fig. 1:**
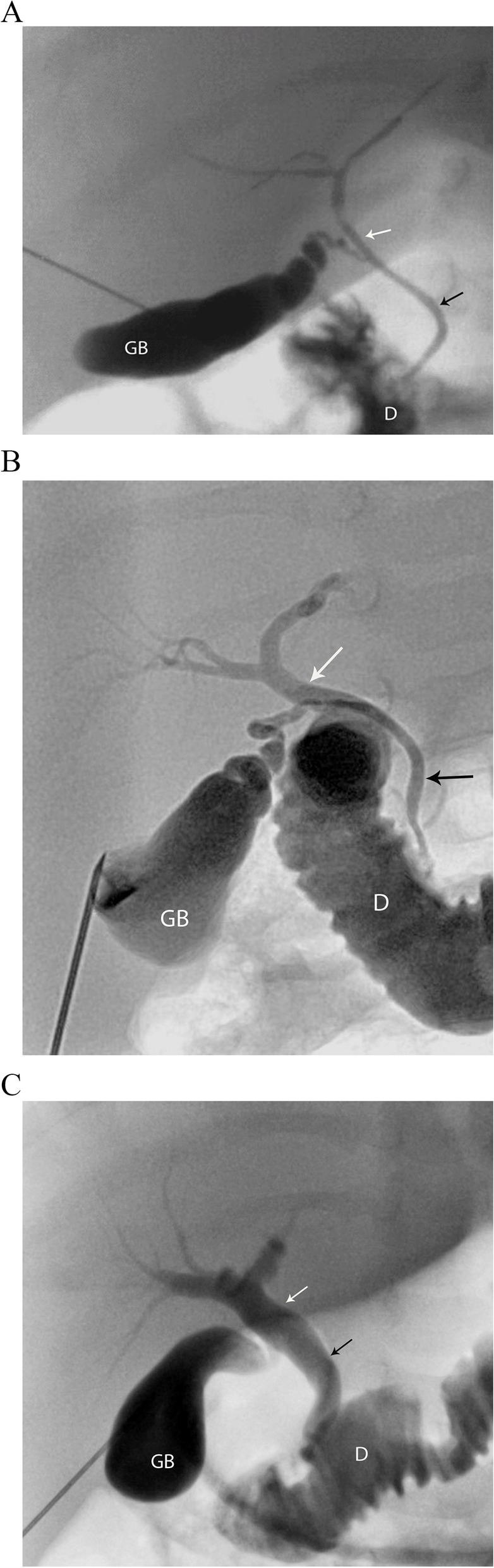
Three cases to illustrate the findings of a normal PTCC in: **(A)** 9-week-old male, 4.4 Kg, final diagnosis of neonatal hepatitis; **(B)** 8-week-old male, 2.3 kg, final diagnosis of neonatal hepatitis and **(C)** 24-week-old female, 5.8 Kg, final diagnosis of PFIC type 1. The gallbladder (GB), the common bile duct (black arrow), the common hepatic duct (white arrow) and right and left hepatic ducts show a normal appearance and passage of contrast into the duodenum **(D)**

Informed consent is obtained from parents after explaining the procedure and associated risks. The procedure is performed under general anesthesia, using sterile technique. Prophylactic antibiotics are given. The gallbladder (GB) (often atretic, small or rudimentary) is localized using US (8 MHz curvilinear or 15 MHz liner probe). Percutaneous trans-hepatic access into the GB is obtained under direct sonographic visualization, using a fine needle (27 Gauge (G), 25G or 22G Chiba, operator’s preference). Access along the GB long axis is preferred but can be obtained transversely (short axis). Successful intra-luminal needle position, with complete mucosal puncture can be confirmed by injecting a small volume of normal saline under US visualization (this avoids inadvertent submucosal or extra-luminal contrast injections). Once needle position is confirmed, a small volume of iodinated contrast (Omnipaque 300, full strength or diluted according to the operator’s preference) is gently and slowly injected while adequately magnified fluoroscopic images are obtained in different planes [Fig. 1].

After observing contrast filling of the common bile duct (CBD) and contrast passage into the duodenum, the injection pressure is gently increased to attempt retrograde opacification of the common hepatic duct (CHD) and proximal ducts. Once completed, residual GB contrast is aspirated, and the GB and bile ducts can then, if needed, be washed out with normal saline.

If requested, an US guided percutaneous core needle liver biopsy may be performed after the PTCC, with or without embolization of the biopsy tract. This embolization is achieved with Gelfoam slurry or with the Hunter biopsy sealing device (Teleflex, Morrisville, NC, USA). Compression may be applied at the puncture site to ensure hemostasis.

The patient is transferred to the post anesthetic care unit and then to the ward for monitoring of clinical condition and vital signs. A complete blood count is obtained 4–6 hours after the procedure. The patient is admitted for a minimum of 24 hours of observation.

### Imaging analysis

A PTCC was reported as normal if the opacified GB appeared normal in size and shape, with prompt contrast passage into the cystic duct, common bile duct (CBD) and duodenum, with concomitant reflux into the common hepatic duct (CHD) and right and left hepatic ducts. A normal GB shows rapid distention, a “pear-like” shape, smooth mucosal contour, a short axis diameter at the distal body the same length or larger than the height of the closest lumbar vertebral bodies, and a long axis three times or longer than the short axis (Note: the normal newborn gallbladder is 1.5 to 3.5 cm in length (17,18,19,20,21), but size was not directly measured on PTCC images). As the primary purpose of the PTCC is to determine the presence and patency of the bile ducts, mild caliber variations seen in the ducts are usually inconsequential. Some contrast may reflux into central intrahepatic ducts, and the pancreatic duct is occasionally opacified [Fig. 1].

A PTCC was reported as abnormal if the opacified GB appeared abnormal in size or shape; if there was non-opacification or abnormal caliber of the cystic duct, CBD, CHD, and if there was lack of contrast passage into the duodenum. Different patterns of opacifications were grouped, categorized, and described in terms of presence or absence of duct opacification. Duct caliber was not measured.

## Results

### Patient cohort

A total of 89 patients were referred for a PTCC during the study period. The procedure was successful in 73 patients, who formed the study cohort. The mean patients’ age at the procedure time was 8.7 weeks (median:8, range: 3–24 weeks) and the mean weight was 4.0 Kg (median 3.9 Kg, IQR: 1.4). 50 (68%) were male. Their final diagnoses after correlation of clinical, radiological, pathological, and surgical findings are illustrated in Table 1. In 16 patients the PTCC was not technically feasible due to the absence of any GB or rudimentary structure for puncture and these 16 cases were excluded from this study (12/16 the final diagnosis was BA).

**Table 1:** Clinical diagnoses of the cohort of patients and PTCC findings (normal/abnormal)

Final Diagnosis	Number of Patients (%)	Normal PTCC	Abnormal PTCC
Biliary Atresia	26 (36%)	0	26
TPN Cholestasis	15 (21%)	15	0
Neonatal Hepatitis	12 (16%)	12	0
Alagille Syndrome	4 (5%)	0	4
Progressive Familial Intrahepatic Cholestasis (PFIC)	4 (5%)	3	1
Williams Syndrome	1 (1%)	1	0
North American Indian Childhood Cirrhosis (NAICC)	1 (1%)	1	0
Alpha-1 Antitrypsin Deficiency	1 (1%)	0	1
G6PD Deficiency	1 (1%)	1	0
Hepatitis of Unknown Origin	8 (11%)	7	1
Total	73	40	33

### Pre-procedure diagnostic US and HIDA scan

All patients had at least one pre-procedure diagnostic US study. The GB was visualized in 71/73 patients (97%). A normal GB was noted in 30/71, and a “small” or “contracted” gallbladder reported in 41/71. The GB descriptions were based on the radiological report, and for this study small and contracted GBs were considered equivalent representing a GB with smaller dimensions than expected. In three cases a small gallbladder was associated with a cyst. In the two cases with no visualization of the GB (2/73) on diagnostic US, a small cyst was visualized in one, and the GB was reported as absent in the other, however, cystic structures were encountered in both patients at the time of their PTCC, obtaining a successful puncture and opacification of these cystic structures.

A HIDA scan was performed in 52 patients (47/52 prior to PTCC). In 41/52 (79%) studies there was no drainage into the duodenum at 24 hours, yet in 22/41 (54%) of these patients the PTCC was normal. The remaining 11/52 HIDA scans were reported as non-conclusive (9), minimal excretion (1) and delayed excretion (1). In 10/11 cases they were associated with a normal PTCC. In patients with BA (*n* = 26), 17/26 patients had HIDA scans performed: 16/17 showed no drainage of isotope into the duodenum and 1/17 (whose study was performed in another center) was reported as “inconclusive”.

### Procedure details

A 27 G needle was used in 37 procedures (51%); a 25G needle in 28 procedures (38%), and a 22G Chiba needle in 8 procedures (11%). A biopsy was performed following the PTCC at the same procedure in 60/73 patients (82%), 8/73 (11%) had undergone a previous percutaneous biopsy and 5/73 (7%) did not undergo any biopsy. A co-axial technique with biopsy tract embolization was used in 35/60. The mean number of passes and mean number of cores were 2.

There were two major complications related to the interventional procedure (SIR D). One patient had a Gram-negative sepsis 1 day after the study, which was successfully treated with antibiotics. The other patient had a bleed associated with free peritoneal fluid on US, a 23 g/L hemoglobin drop which required blood transfusion, and the patient recovered. US showed the presumed source was at the biopsy site, rather than the PTTC. There was one minor complication (SIR A) involving a patient with transient self-limited abdominal distension, without abnormal findings on US. Two complications unrelated to the PTCC were seen: One difficult intubation which required 5 days of NICU admission for recovery, and one subcapsular liver hematoma related to the biopsy site, which did not require blood transfusions or other interventions.

### PTCC findings

The PTTC biliary tree appearance was normal in 40/73 (55%) patients, and their final diagnoses are shown in Table 1. The most common etiologies were TPN-related cholestasis and idiopathic neonatal hepatitis [Fig. 1]. The pre-procedure diagnostic US in these 40 infants showed a normal GB in 29/40 (73%) cases, a contracted GB in 9/40 (22%) and a small GB in 2/40 (5%).

**Fig. 2:**
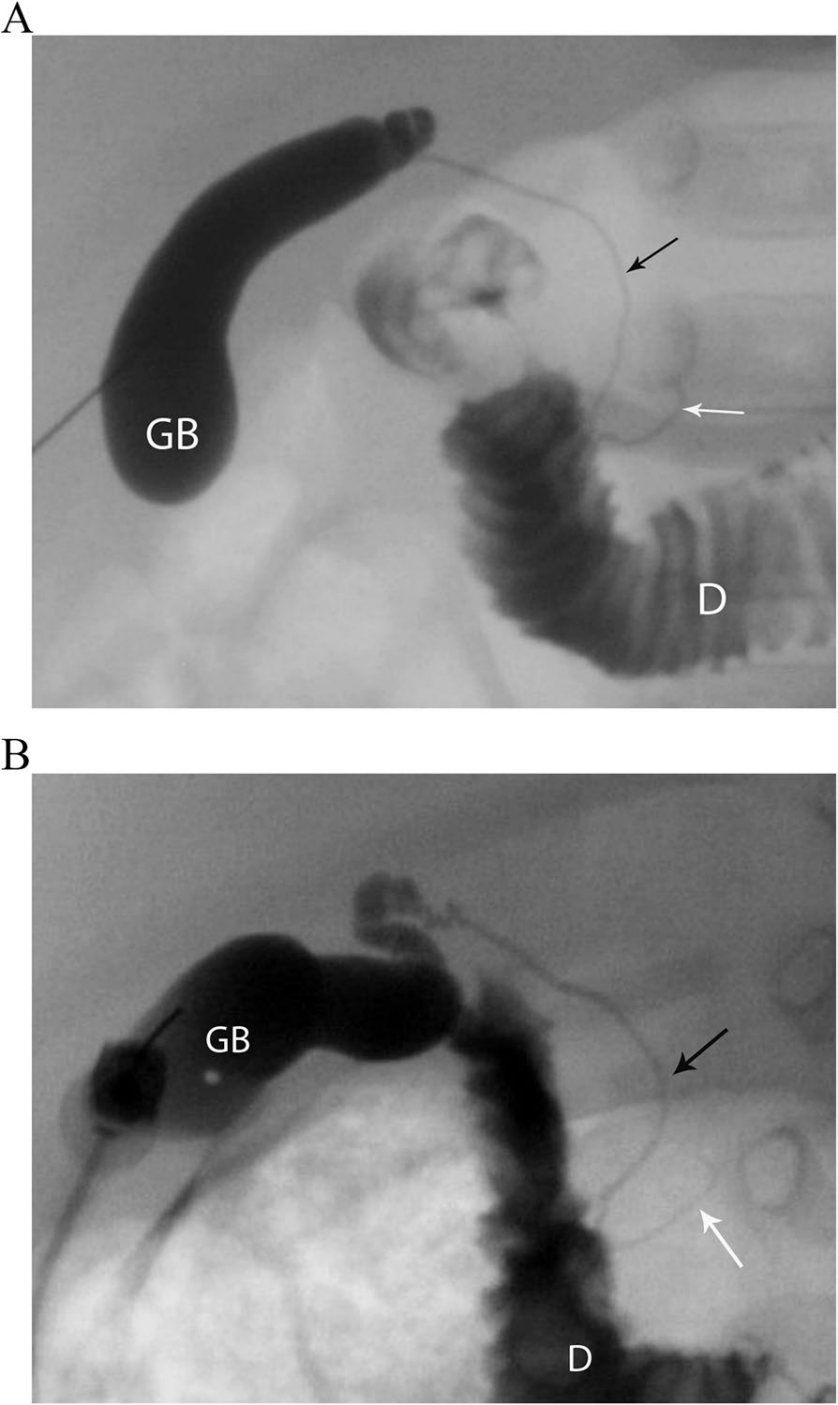
Two cases of BA showing non opacification of the common hepatic duct and a narrowed common bile duct (black arrow). The gallbladder (GB) is present, there is passage of contrast into the duodenum (D) and pancreatic duct opacification (white arrow). Both patients underwent a Kasai procedure, however eventually required an orthotopic liver transplant. **(a)** 7-week-old male, 4.9 Kg; **(b)** 3-week-old male, 5.9 Kg

An abnormal PTCC was found in 33/73 (45%) patients, and their final diagnoses are shown in Table 1. The majority had BA (26/33; 79%); Abnormal bile duct opacification was found in all 26. No patient subsequently diagnosed as BA had a normal PTCC.

**Fig. 3:**
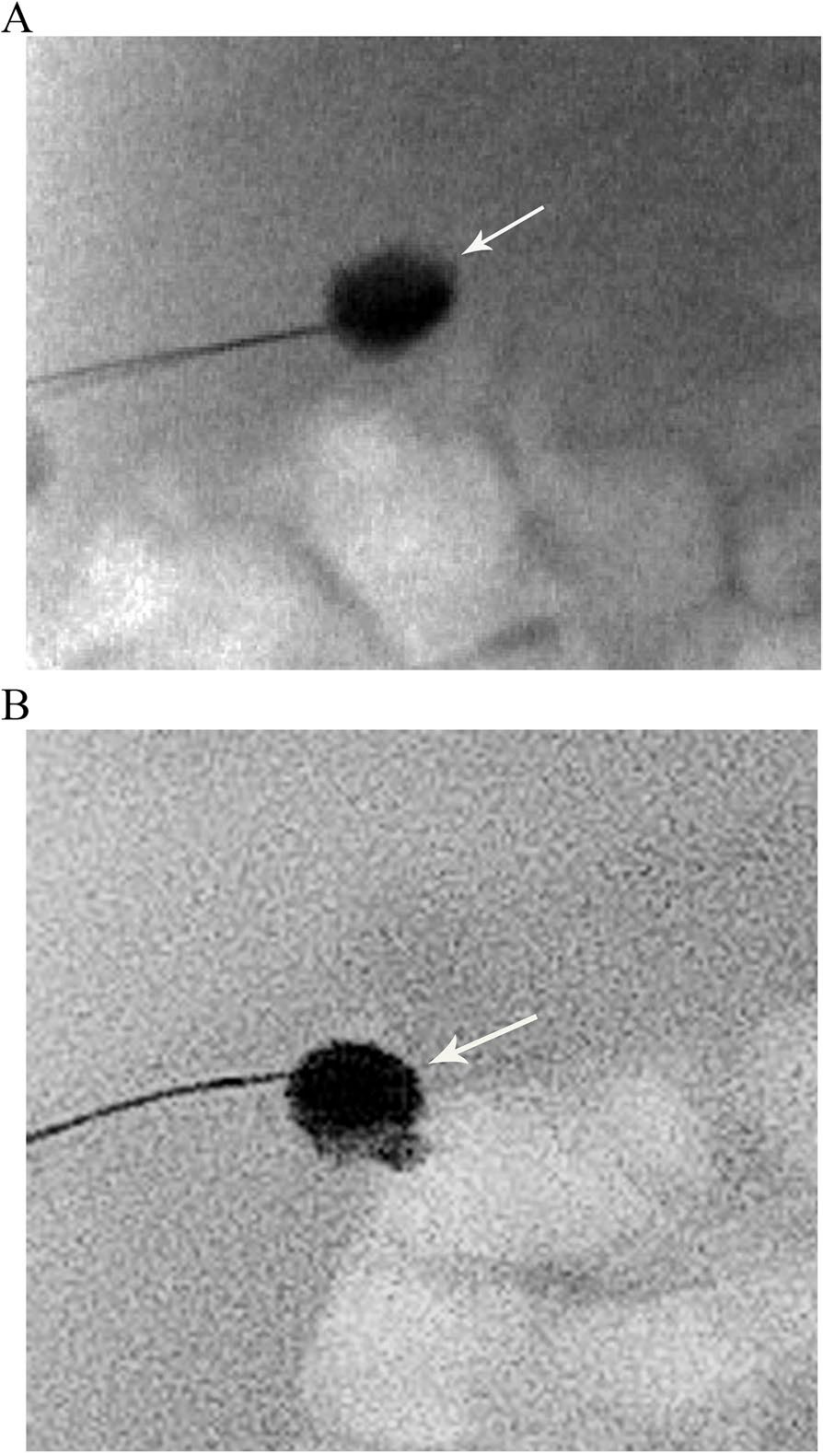
Two cases of BA with an isolated small gallbladder (arrow) with lack of opacification of the reminder of the biliary tree. After a Kasai procedure, both patients required an orthotopic liver transplant. **(a)** 8-week-old female, 4 Kg; **(b)** 16-week-old male, 6 Kg

**Fig. 4:**
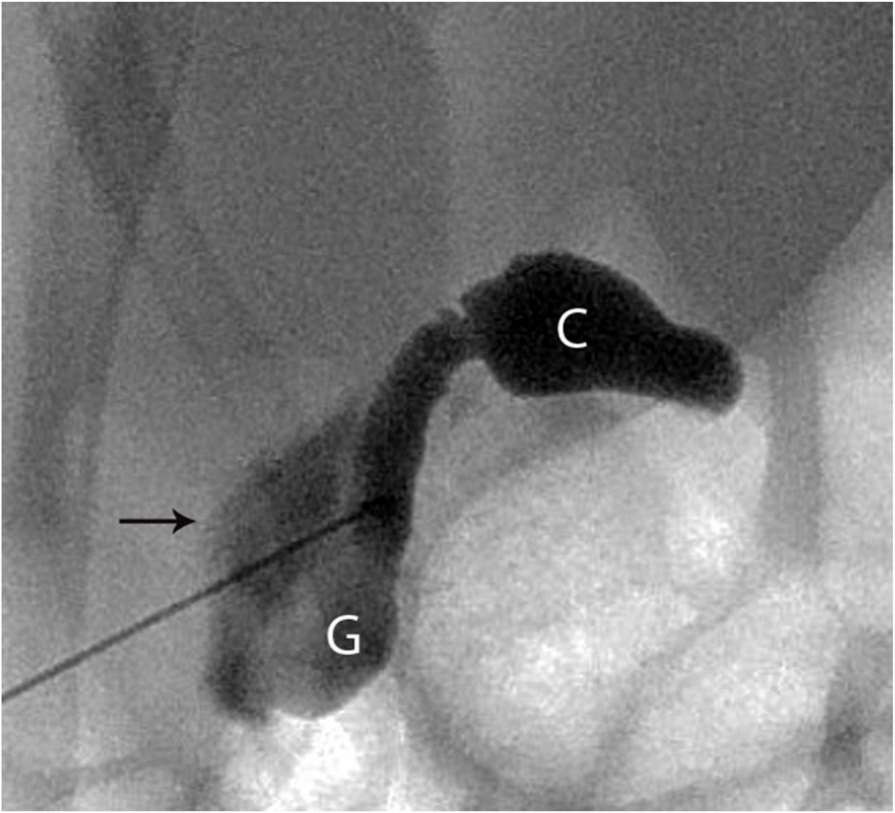
A case of cystic BA in a 5-week-old male, 4.4 Kg. A small gallbladder (G) communicated with a cyst (C) was observed. No opacification of the reminder of the biliary system. A small amount of contrast extravasation is noted (arrow). The patient underwent a Kasai procedure

**Fig. 5:**
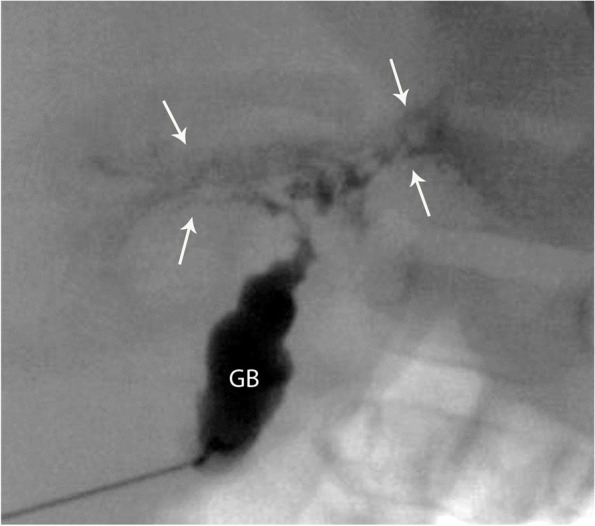
A 10-week-old female, 5.5 Kg with Pierre Robin sequence and final diagnosis of BA. PTCC showed a small abnormally shaped gallbladder (GB), connected to a short common hepatic duct and opacification of intrahepatic ducts (arrows). No opacification of the common bile duct or passage of contrast into the duodenum was demonstrated. A Kasai portoenterostomy was performed

Among those with BA, different PTCC patterns were encountered:

1. Non-opacification of CHD and narrowed CBD: Characterized as thin appearing cystic duct and CBD and variable GB size. No opacification of CHD or right and left hepatic ducts observed. Relatively slow and low volume of contrast passage into the duodenum. This was the most common abnormal PTCC pattern, observed in 11/26 cases (42%) [Fig. 2]. The pre-procedure diagnostic US showed a small GB in 10/11, and a normal GB in 1/11.
2. Isolated small GB with no opacification of the remainder of the biliary tree: The opacification of a small cystic structure which did not communicate with any bile ducts was described as an isolated small GB. This was the second most common abnormal finding, seen in 10/26 cases (38%) [Fig. 3]. The pre-procedure diagnostic US reported a “small” gallbladder in 8/10, and an “absent” GB and a “small cyst” in the remaining 2/10 patients.
3. Cystic Biliary Atresia: Opacification of a small GB communicating with a blind-ended cyst was observed in 3/26 cases (12%). The cysts were of variable size and there was no communication with bile ducts or bowel [Fig. 4]. The pre-procedure diagnostic US showed a small GB connected with a cyst in all three cases.
4. Small GB with non-opacification of CBD and abnormal intra-hepatic ducts (IHD): Opacification of a small GB connected to irregular and thin IHD was seen in 2/26 cases (8%) [Fig. 5]. A small GB was seen on the pre-procedure diagnostic US.

**Fig. 6:**
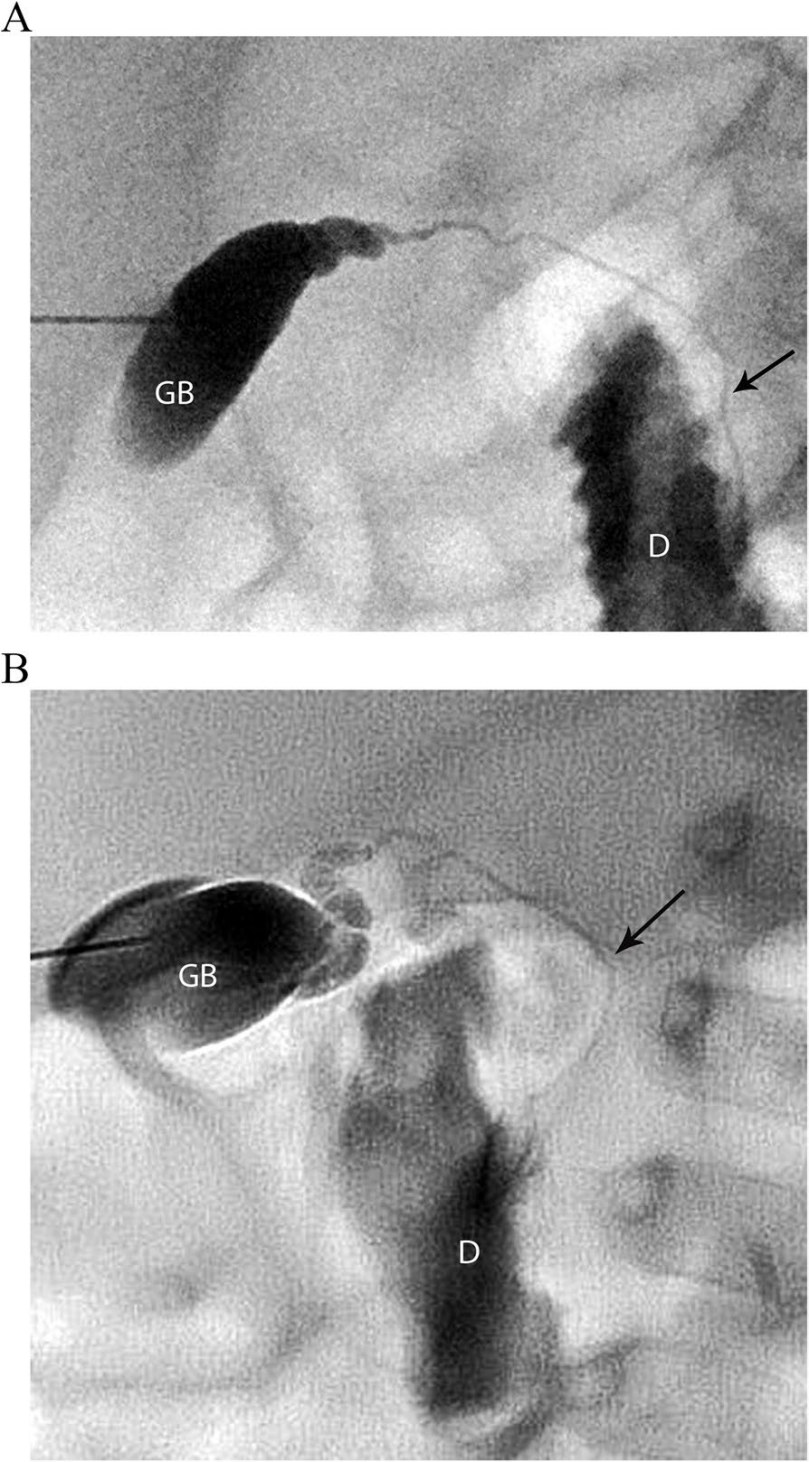
Two patients with final diagnosis of Alagille syndrome in whom the PTCC showed a gallbladder (GB), lack of opacification of the common hepatic duct, narrowed common bile duct (arrow) and passage of contrast into the duodenum (D). **(a)** 12-week-old male, 6.5 Kg **(b)** 6-week-old female, 3.1 Kg. Both patients were managed medically

Among the remaining 7/33 patients with abnormal PTCC patterns, four (4/33; 12%) were diagnosed with Alagille syndrome and all of them had non-opacification of the CHD and narrowed CBD pattern [Fig. 6].

**Fig. 7:**
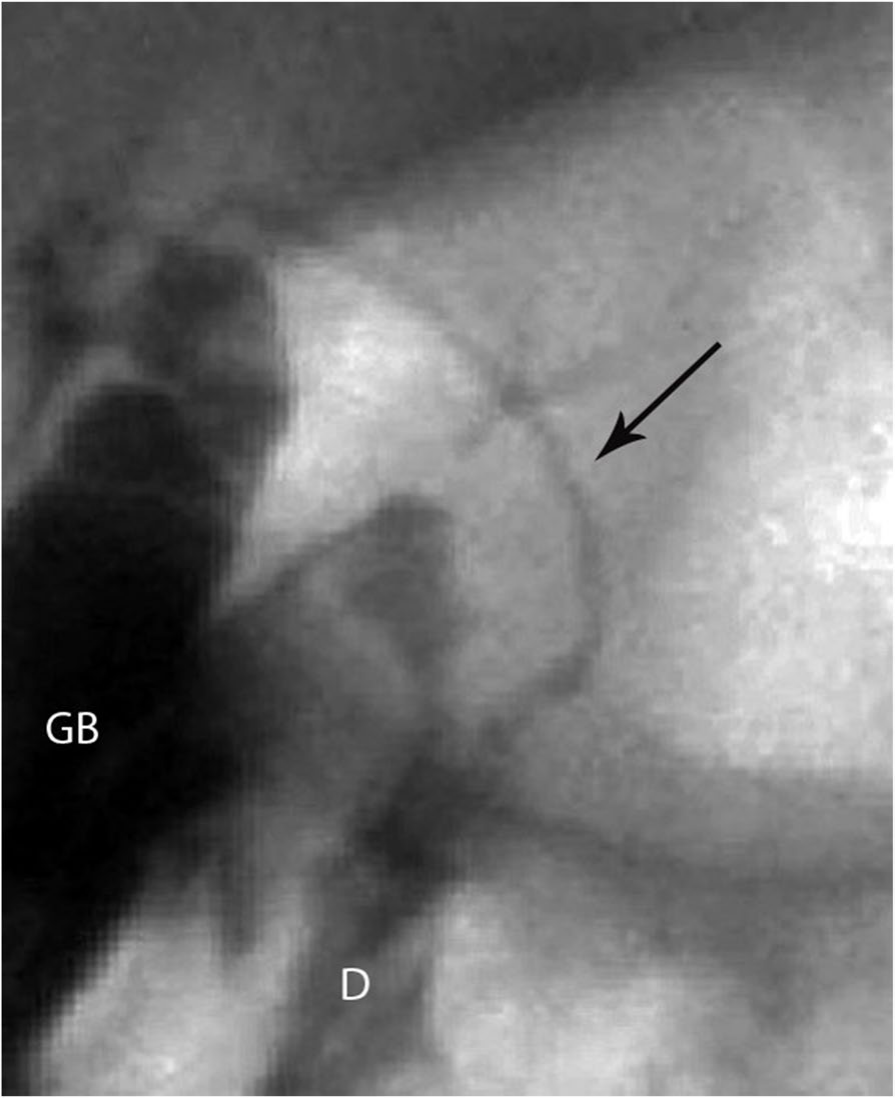
A 10-week-old male, 4.5 Kg, presenting with conjugated hyperbilirubinemia, jaundice and acholic stools. The final diagnosis was Alpha 1 Antitrypsin deficiency. The PTCC showed a gallbladder (GB), lack of opacification of common hepatic duct, narrowed common bile duct (arrow) and passage of contrast into the duodenum (D). The patient received an orthotopic liver transplant

Alpha-1 antitrypsin deficiency showed a similar pattern in 1/33 (3%) [Fig. 7].

**Fig. 8:**
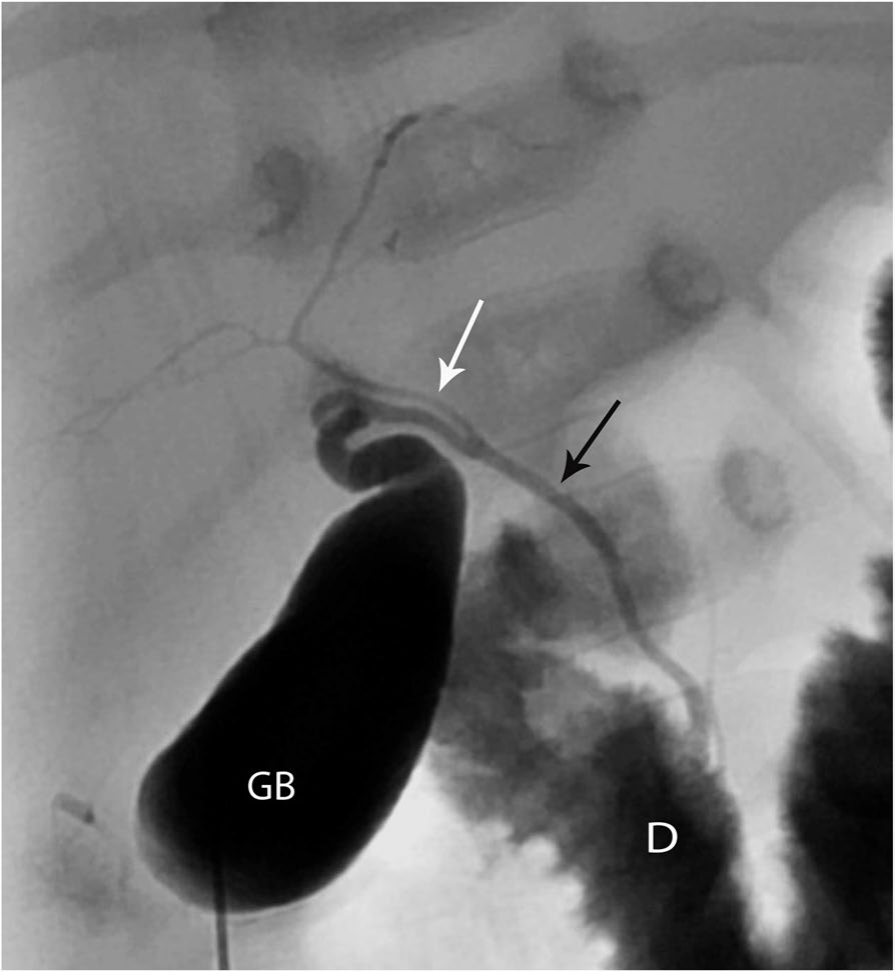
A 3-week-old male, 3.5 Kg, referred for conjugated hyperbilirubinemia. The PTCC showed a relatively thin common hepatic duct (white arrow) in comparison to the common bile duct (black arrow), with passage of contrast into the duodenum (D) and normal gallbladder (GD). The patient recovered normal liver function with medical management and no other interventions were required

In 2/33 (6%) a relatively thin CHD compared to the CBD was seen – one was diagnosed with Progressive Familial Intrahepatic Cholestasis (PFIC), while the second recovered normal liver function [Fig. 8].

These seven patients had a small GB seen in the pre-procedure diagnostic US. Table 2 summarizes the different PTCC patterns and their corresponding diagnoses.

**Table 2:** PTCC patterns and corresponding diagnoses

PTCC pattern	Diagnosis (n)
Non-opacification of CHD and narrowed CBD	Biliary Atresia (11)
	Alagille Syndrome (4)
	Alpha-1 Antitrypsin Deficiency (1)
Isolated small GB with no opacification of the remainder of the biliary tree	Biliary Atresia (10)
Cystic Biliary Atresia	Biliary Atresia (3)
Small GB with non-opacification of CBD and abnormal intra-hepatic ducts	Biliary Atresia (2)
Relatively thin CHD compared to the CBD	Progressive Familial Intrahepatic Cholestasis (PFIC) (1)
	Hepatitis of Unknown Origin (1)
Normal PTCC	TPN Cholestasis (15)
	Neonatal Hepatitis (12)
	Progressive Familial Intrahepatic Cholestasis (PFIC) (3)
	Williams Syndrome (1)
	North American Indian Childhood Cirrhosis (NAICC) (1)
	G6PD Deficiency (1)
	Hepatitis of Unknown Origin (7)

*PTCC* Percutaneous Trans-Hepatic Cholecysto-Cholangiography, *CHD* Common Hepatic Duct, *CBD* Common Bile Duct, *GB* Gallbladder.

### Surgery

A Kasai procedure was performed in 24/26 patients with BA. In the remaining 2 patients an orthotopic liver transplant (OLT), without prior Kasai, was required due to late presentation and advanced cirrhosis. Of the 24 patients who had a Kasai, 11 subsequently had an OLT. Three additional patients underwent an OLT during the study period, for Alagille syndrome, North America Indian childhood cirrhosis (NAICC) and progressive familial intrahepatic cholestasis (PFIC) type 1.

### Laboratory values

A Mann-Whitney U test indicated that GGT levels were significantly higher in those with abnormal PTCC patterns (median = 544, mean rank: 43.75) compared to normal PTCC patterns (median = 213, mean rank: 25.09) U = 248, *p* < 0.05. Similarly, GGT was significantly higher in patients diagnosed with BA (median = 614, mean rank: 47.10) compared to other diagnoses (median = 205, mean rank:31.79), U = 252, p < 0.05. The difference in total bilirubin, AST, ALT and GGT between patients diagnosed with BA and Alagille syndrome was not significant.

## Discussion

This study demonstrated the variety of findings seen on PTCC in a relatively large patient cohort with suspected BA. 55 studies were normal, and thus avoided further invasive investigations requiring general anesthesia and laparotomy in these young patients. The most common diagnosis in those with an abnormal PTCC was BA; the two most common patterns were i) failure to opacify the CHD associated with a narrowed CBD, and ii) an isolated small GB. The second most common diagnosis was Alagille syndrome, all of whom demonstrated patent but narrowed CBD and lack of CHD opacification.

Percutaneous transhepatic cholecysto-cholangiography in this center is considered part of the diagnostic work-up of neonates and infants presenting with conjugated hyperbilirubinemia, especially those suspected of BA. It is a minimally invasive alternative to the intra-operative cholangiography and histologic examination of the duct remnants of the porta hepatis, which are considered the standard criterion for the diagnosis of BA [3, 6–9]. It is crucial to practice careful technique, using high quality imaging and proper intraluminal GB puncture, to prevent contrast extravasation, which obscures visualization and can compromise procedural success. There is no minimal patient weight or size for this procedure, the only requirement is to have a GB or cystic structure that can be punctured. Successful training can be achieved in centers with high volume of these procedures, usually by dedicated pediatric interventional radiologists. Identification of a normal biliary tree appearance and drainage during diagnostic work-up is of major importance as it obviates the need for surgery [10–13, 17].

The normal length of the GB in the first year of life has been described to be approximately 1.5 to 3.5 cm [18–21], however it can be difficult to differentiate between a small and a contracted GB. The spectrum of US findings in BA includes a small, absent or an atretic GB without a lumen [21]. A collapsed or non-visualized GB on US is found in 50–60% of BA patients [16, 22]. In the group with normal PTCC, 73% of the patients had a normal GB on their pre-procedure diagnostic US, the remainder had a small or contracted GB, therefore a small or contracted GB is not a contraindication for a PTCC and can be seen in normal studies. The majority of patients (94%) with abnormal PTCC findings had small or contracted GB on pre-procedure diagnostic US, one patient had an absent GB (cyst noted at the procedure day) and one had a small cyst. Only one patient with BA had a normal GB reported in pre-procedure diagnostic US. Based on this, independent of the size, presence, or absence of a GB in the pre-procedure diagnostic US, the GB is re-assessed at the time of the procedure. If nothing is visible to target, a liver biopsy is obtained, or the procedure is aborted. Attempts to access intrahepatic bile ducts are not routinely performed.

When the clinical, laboratory and sonographic findings remain indeterminate, a PTCC is recommended [17]. A PTCC can delineate the anatomy of the biliary system to either exclude BA or help direct further diagnostic steps. Lack of CHD opacification with CBD narrowing is not pathognomonic for any condition, as it may be associated with BA or hypoplasia [Figs. 2, 6,7]. 16 cases had this pattern, 11 of whom (61%) were diagnosed with BA. However, the pattern was also found in biliary hypoplasia/Alagille syndrome and Alpha-1 antitrypsin deficiency. Similar findings with Alagille syndrome have been found by others [16].

A morphologically abnormal GB associated with a non-communicating cyst in either an intra-hepatic or extra-hepatic location is most consistent with cystic BA. Cystic BA was seen in 12% of BA cases, which is consistent with previous reports [23]. The main differential diagnosis of this condition is a choledochal cyst, which can be associated with dilated intra-hepatic bile ducts, a larger cyst, and normal appearing GB [23].

The presence of normal opacification and morphology on PTCC makes the diagnosis of BA very unlikely, and helps avoid further invasive investigations, such as laparotomy with intraoperative cholangiography, in most patients. These results are consistent with previous studies which have identified up to 72.2% reduction of other invasive diagnostic procedures using PTCC [10, 16]. On later follow-up, none were subsequently diagnosed as BA. In our practice, a normal PTCC does not change the decision to perform a planned liver biopsy during the same visit to IR as histologic assessment will aid in the differential diagnosis of the ongoing cholestasis.

The GGT levels were significantly higher in patients with an abnormal PTCC compared to a normal PTCC. In jaundiced neonates and infants presenting with an elevated GGT, an abnormal cholangiography may be likely. High GGT cholestasis has been described in Alagille syndrome, PFIC type 3 and frequently in BA [3]. In the diagnosis of BA, elevated GGT has diagnostic accuracies at varying cut-off levels, and is particularly useful in patients < 120 days old; it is incorporated into diagnostic work-up for BA [17, 24, 25]. In this study the levels of GGT were significantly higher in patients diagnosed with BA compared to other diagnoses. This study’s results further support the use of GGT in the diagnostic work-up of suspected BA.

There are several limitations to this study. This was a retrospective study, and occasional suboptimal patient records or imaging reports were found. The different PTCC findings were not quantified or calculated, which makes it difficult to define normal measurements. With new equipment and IR technology, it is feasible to perform measurements on visualized structures and potentially establish normal parameters – this could be the focus of future studies. It is possible that some BA cases with abnormal or absent GB on diagnostic US, were not included as they may have proceeded directly to surgery, therefore, patients with narrowed CBD, as opposed to other patterns may be over-represented in this study’s cohort. The differentiation between a contracted and small GB in the pre-procedure diagnostic US can be confusing. This terminology was quoted verbatim from radiological reports by a large cohort of diagnostic radiologists and is based on their interpretation of the images and not necessarily on any absolute difference in measurements, however most of the time both terms described a GB less than 1.5 cm in length. Radiation dose metrics were not available for many of the PTTC studies. The total number of patients presenting with conjugated hyperbilirubinemia or the total number of cases with BA during the study period were not determined; an earlier study over 10 years of observation found 212 and 35 patients respectively [5]. Lastly, there may be potential inaccuracies when collating patients’ diagnoses and treatment outcomes from clinical, pathological, and radiological data.

In conclusion, PTCC is a minimally invasive imaging procedure to assess the biliary system in neonates with hyperbilirubinemia and suspected BA. With increasing frequency, it is added to the diagnostic work-up of this condition [3, 16, 17, 26, 27]. It is especially valuable when normal, as it can avoid unnecessary surgery. This paper describes in a large patient cohort abnormal patterns that warrant further investigations, with a low procedure related complication rate. It is important for the pediatric (interventional) radiologist performing this procedure, the pediatric surgeon and pediatric hepatologist, to be familiar with the different patterns encountered, and to collaborate meaningfully in the management of these patients.

## Data Availability

The data that support the findings of this study are available from The Hospital for Sick Children, but restrictions apply to the availability of these data, and so are not publicly available. Data are however available from the authors upon reasonable request and with permission of The Hospital for Sick Children.

## Abbreviations

BA: Biliary atresia
PTCC: Percutaneous Trans-hepatic Cholecysto-Cholangiography
US: Ultrasound
PN: Parenteral nutrition
PNALD: Parenteral nutrition-associated liver disease
HIDA: Hepatobiliary Imino-Diacetic Acid
EPC: Electronic Patient Chart
PACS: Picture and Archiving Communication System
AST: Aspartate Aminotransferase
ALT: Alanine Aminotransferase
GGT: Gamma-Glutamyl Transferase
GB: Gallbladder
CBD: Common Bile Duct
CHD: Common Hepatic Duct
IHD: Intra Hepatic Bile Ducts
PFIC: Progressive Familial Intrahepatic Cholestasis
EHBA: Extrahepatic BA
SIR: Society of Interventional Radiology
IQR: Interquartile range
